# Complete Genome Sequences of *Helicobacter pylori* Rifampin-Resistant Strains

**DOI:** 10.1128/genomeA.00446-13

**Published:** 2013-07-05

**Authors:** Kuvat Momynaliev, Vera Chelysheva, Oksana Selezneva, Tatyana Akopian, Dmitry Alexeev, Vadim Govorun

**Affiliations:** National Center for Biotechnology, Astana, Kazakhstana; Research Institute for Physico-Chemical Medicine, Moscow, Russiab

## Abstract

Here we present the complete genome sequences of two *Helicobacter pylori* rifampin-resistant (Rif^r^) strains (Rif1 and Rif2). Rif^r^ strains were obtained by *in vitro* selection of *H. pylori* 26695 on agar plates with 20 µg/ml rifampin. The genome data provide insights on the genomic diversity of *H. pylori* under selection by rifampin.

## GENOME ANNOUNCEMENT

*Helicobacter pylori* is a Gram-negative, microaerophilic, helix-shaped bacterium that colonizes the stomach of at least half of the world’s human population (1, 2). In most cases, *H. pylori* can persist in the human stomach without health consequences, though it is a risk factor for chronic gastritis, gastric or duodenal ulcers, and gastric cancer (3, 4). Treatment to eliminate *H. pylori* infection may fail when antibiotic-resistant strains are present.

In the present report, we announce the availability of two genome sequences of *Helicobacter pylori* rifampin-resistant (Rif^r^) strains (Rif1 and Rif2). The genome data provide insights on the genomic diversity of *H. pylori* under selection by rifampin.

Rif^r^ strains were obtained by *in vitro* selection of *H. pylori* 26695 on agar plates with 20 µg/ml rifampin and were sequenced on a Roche GS FLX genome sequencer using a standard protocol for a shotgun genome library. Sequencing rounds yielded approximately 351,807 sequences with an average read length of 109 bases for the *H. pylori* Rif1 strain (23-fold coverage) and 375,434 sequences for *H. pylori* Rif2 (25-fold coverage). The GS FLX reads were assembled into contigs by a GS De Novo Assembler (Roche). The contigs were oriented into scaffolds, and the complete genome sequence was obtained upon the generation and sequencing of appropriate PCR fragments. Sequences were annotated with the NCBI Prokaryotic Genomes Automatic Annotation Pipeline (5).

In the two Rif^r^ strains, 11 and 9 mutations (including mutations in the *rpoB* gene) were found. Five mutations (T389863С, insertion of T at position 597760, G748722T, T1189153G, and deletion of GA at position 1487684) were common to both strains. Six mutations were unique to the *H. pylori* Rif1 strain, including mutations in the *rpoB* gene (T588I [G1275287A], D530N [C1275462T], and S155I [C1276586A]), and four were unique to *H. pylori* Rif2, including one mutation (H540Y [G1275432A]) in the *rpoB* gene.

### Nucleotide sequence accession numbers.

The genome sequences of *Helicobacter pylori* rifampin-resistant (Rif^r^) strains Rif1 and Rif2 were deposited at GenBank under the accession numbers CP003905 and CP003906, respectively.
